# New Species of *Entoloma* Subgenera *Cubospora* and *Leptonia* (Agaricales, Basidiomycota) from Central Vietnam

**DOI:** 10.3390/jof9060621

**Published:** 2023-05-27

**Authors:** Olga Morozova, Thi Ha Giang Pham

**Affiliations:** 1 Komarov Botanical Institute of the Russian Academy of Sciences, 2 Prof. Popov Str., 197022 Saint Petersburg, Russia; 2 Joint Vietnam-Russia Tropical Science and Technology Research Centre, Nguyen Van Huyen, Nghia Do, Cau Giay, Hanoi 122100, Vietnam

**Keywords:** taxonomy, systematics, Entolomataceae, molecular phylogeny, new species, Kon Chu Rang Nature Reserve, Ta Dung National Park

## Abstract

Four new species of *Entoloma* from Kon Chu Rang Nature Reserve and Ta Dung National Park were discovered during an investigation of the diversity of the mycobiota of Central Vietnam and are described here on the base of the molecular and morphological data. Phylogenetic analysis was based on nrITS1-5.8S-ITS2, nrLSU and *tef1α* regions. Illustrated descriptions of their macro- and microscopic features and discussion on similar taxa are given. *Entoloma cycneum* and *E. peristerinum* belong to the subgenus *Cubospora*. They are morphologically similar species and are characterized by white or whitish basidiomata with yellowish or beige tinges and with mainly smooth, glabrous, and hygrophanous pileus, longitudinally fibrillose or fibrillose-scaly white stipe, cuboid spores, and more or less cylindrical cheilocystidia, arising from hymenophoral trama. *Entoloma peristerinum* posseses initially more coloured beige conical pileus, discolouring to white with age and drying. The pileus of *E. cycneum* is initially white, hemisphaerical to convex, usually with thin pubescence near the margin. The species can be recognized also by the cheilocystidia form: serrulatum-type in *E. cycneum* vs. porphyrogriseum-type in *E. peristerinum.* Another two species belong to the subgenus *Leptonia*. *Entoloma tadungense* is close to *E. percoelestinum* from which it differs by smaller spores with pronounced angles, presence of the cheilocystidia, and the lilac discolouration of the stipe. *E. dichroides* is named after its similarity to *E. dichroum*, a dark blue coloured species with pronouncedly angled basidiospores. It is distinguished by the basidiospores form—irregularly 5(–6) angled with elongated apiculus, as well as by absence of the cheilocystidia and darker basidiomata with conical pileus. The article also describes the history of the study of the genus *Entoloma* in Vietnam with a list of 29 species mentioned in the publications for this country.

## 1. Introduction

According to estimates for 2018 [1], 21 species of the genus *Entoloma* (Fr.) P. Kumm. were known from the Central Vietnam, including unpublished data of the authors. In subsequent years, five more new species were described from this territory [2,3,4]. The full history of study of the genus *Entoloma* in Vietnam with a list of 29 species mentioned in the publications for this country is described in the discussion part. However, the real diversity of the genus is much higher, and many species are still waiting to be described.

As proven by recent molecular genetic studies, the shape of the basidiospores is a key feature in understanding evolution and relatedness in Entolomataceae [5,6,7].

The cuboid and cuboid-like form of spores is remarkable and interesting from a phylogenetic point of view in terms of the multiplicity of its occurrence in the course of evolution. The molecular genetic data [7] supports a clear segregation of clades with cuboid spores from cuboid-like ones (pentagonal, prismatic, pseudocuboid, squamiferum). The species with rhomboid spores also cluster separately in a well-supported clade but they are mixed with species with five or six angled spores [8,9].

Species with “true” cuboid spores (with six, more or less equal, quadrangular faces) traditionally were included in the subgenus/genus *Inocephalus* [*Entoloma* subgen. *Inocephala* Noordel., *Inocephalus* (Noordel.) P. D. Orton.]. However, the type of *Inocephalus*, *Entoloma inocephalum* (Romagnesi) Dennis, described from Madagascar, has 5–7 angled basidiospores in profile view [10]. The material identified as *Entoloma inocephalum* from Vietnam [11] has the same morphology and is used as a reference collection for the phylogenetic studies while authentic or Madagascar-collected material is not available [7]. It turns out that the /Inocephalus clade occupies an isolated position from the clades with cuboid spores. Therefore, two new subgenera were described to arrange species with such spores—*Entoloma* subgenus *Cubospora* Karstedt, Capelari, Largent, T. J. Baroni & Bergemann with *E. luteolamellatum* (Largent & Aime) Blanco-Dios as a type species (with a conical pileus) and *Entoloma* subgenus *Cuboeccilia* Karstedt, Capelari & Largent with *E. omphalinoides* (Largent) Blanco-Dios as a type species (with depressed pileus) [7]. The number of species with cuboid and cuboid-like spores described world-wide so far is estimated to be 165 [12] or 120 [7].

The first monographic contributions devoted to groups with cuboid spores were made by Horak [13,14,15,16,17], and refer to SE-Asia and South America. The following publications deal with the diversity of cuboid-spored species in different regions, e.g., Asia [11,12,18,19,20,21], Africa/Madagascar [10,22,23,24], Australia/Tasmania/New Zealand [25,26], South and Central America [27,28,29,30], and North America [31,32]. Generalization of accumulated data based on phylogenetic analysis was conducted by F. Karstedt et al. [7]. The analysis of records shows that the centre of diversity of the group with “true” cuboid spores is situated in tropical and subtropical regions of the Old and New Worlds. Two more species of the subgenus *Cubospora* are described from Vietnam here.

Another two new species belong to the subgenus *Leptonia* in the modern sense [8]. The multigene phylogeny based on nrITS, nrLSU, and mtSSU [5] showed that subgenus *Leptonia* in the previously considered volume [32,33,34,35,36] is polyphyletic. Sect. *Leptonia* of the subgenus belongs to the /Nolanea-Claudopus clade, and *Cyanula* and *Griseorubida* to the /Inocephalus-Cyanula clade. Based on these data, *Cyanula* has been raised to the subgenus level [26]. Recent studies [37,38] have shown that a clade of species around *Entoloma ameides* (sect. *Ameides* (Largent) Reschke et al.) previously classified under the subgenus *Nolanea* now is grouped with the subgenus *Leptonia* as a sister to the /Leptonia clade with high support. For that reason, the /Ameides clade is treated as a new section of subgenus *Leptonia* [8]. The subgenus *Leptonia* now combines species with more or less coloured (predominantly with blue, brown, but also yellow or green tinges) basidiomata, characterized also by the presence of clamp-connections, absence of brilliant granules, and more or less fibrillose to squamulose stipe, and includes around 50 species world-wide [8,26,32,39]; Gasteroid forms also occur [37]. The highest diversity of this group was found in the temperate zone of the Holarctic. In the tropics, representatives of the subgenus are rare, and in Vietnam they are recorded for the first time.

## 2. Materials and Methods

### 2.1. Collecting and Site Description

The material for this study was collected during the expeditions of the Joint Vietnam-Russia Tropical Science and Technology Research Centre (VRTC) to the Central Highlands of Vietnam.

The Kon Chu Rang Nature Reserve is located in the north-eastern part of Gia Lai Province of Vietnam (Son Lang Commune, K’Bang District), between 14.50° N–14.58° N and 108.5° E–108.65° E. The area of the reserve is 15,446 hectares, of which 99% are primary and intact forests, which is the highest forest coverage in the country. The relief is hilly to mountainous in the northern part, with heights from 800 to 1452 m a. s. l. (Kon Chu Rang Mt). The average annual temperature is about 21 °C (from 28 °C in May to 12 °C in January). The average annual precipitation is about 1900–2000 mm with the peak in September (340 mm). The dry season is from January to April [40,41]. The main forest type in the reserve is middle-mountain evergreen broad-leaved and mixed forest dominated by Fagaceae (*Lithocarpus*, *Quercus*, *Castanopsis*), Lauraceae, Fabaceae, Clusiaceae, Myrtaceae, Ericaceae, Burseraceae, and Magnoliaceae, mixed with gymnosperms (*Dacrycarpus imbricatus*, *Dacrydium elatum*), distributed at elevations between 900 and 1500 m in the north-west of the nature reserve. The first data on the mycobiota of Kon Chu Rang Nature Reserve were published only recently, including those on several subgenera of *Entoloma*, and on the Boletaceae [3,4,42,43].

Ta Dung National Park is located in the Central Highlands of Vietnam within the administrative boundary of Dak Som commune, Dak G’long district, Dak Nong province, 50 km northeast of Gia Nghia commune’s exam centre. It extends between 11.79° N–11.99° N and 107.89° E–108.11° E, occupying a total area of 20,973.7 hectares. The National Park is located on the Dak Nong Plateau and part of the Di Linh Plateau. Dak Nong Plateau is an arched elevation with an average height of 600–1200 m and has many mountain ranges with an average height of 1200–1500 m, with Ta Dung peak being 1982 m high, the lowest elevation being the land. Ta Dung National Park is situated in an area with a tropical highland monsoon climate regime with two distinct seasons, the rainy season lasts from April to October, the dry season—from November to March. The average annual temperature is 22.0 °C. The total average annual rainfall is 2339 mm; precipitation falls mainly from May to October. Ta Dung National Park is characterized by subtropical humid evergreen closed forests and mixed broadleaf and coniferous closed forests [44]. The mycobiota of the National Park previously was not studied.

Specimens were photographed in the field, and their macromorphological characters, such as size, colour, shape, and surface of all parts of the basidiomata as well as odour, were documented before drying. Colour codes refer to Kornerup & Wanscher [45]. GPS coordinates of collection site, habitat, and substrate type were also documented for each collection. Specimens were then dried either in airtight plastic containers with silica gel, or with an electric dryer at a temperature ca. 50 °C, placed on a piece of absorbent paper and packed in plastic Ziploc bags with small amounts of silica gel.

### 2.2. Morphological Study

Microscopic measurements and drawings were made with an AxioScope A1 light microscope equipped with Zeiss AxioCam 1Cc3 digital camera with AxioVisionRel.4.6 software (CarlZeiss, Jena, Saxe-Weimar-Eisenach, Germany). Spores, basidia, and cystidia were observed in squash preparations of small parts of the lamellae in 5% KOH or 1% Congo Red in concentrated NH4OH. The pileipellis was examined from a radial section of the pileus in 5% KOH. Basidiospore dimensions were based on 20 spores; cystidia and basidia dimensions on at least 10 structures per collection. Basidia were measured without sterigmata, and the spores without apiculus. Spore length to width ratios were reported as Q. When studying the specimens and compiling morphological descriptions, we followed the recommendations and terminology of [8]. The dried specimens were deposited in the Mycological Herbarium of the Komarov Botanical Institute RAS (LE) and in the Herbarium of the Joint Vietnam–Russia Tropical Science and Technology Research Centre, Hanoi (VRTC).

### 2.3. DNA Extraction, Amplification, and Sequencing

PCR products were obtained without DNA purification step using the Thermo Scientific Phire Tissue Direct PCR Master Mix (Thermo Fisher Scientific, Waltham, MA, USA) standard protocol. The ribosomal ITS1–5.8S–ITS2 region was amplified with the fungal specific primers ITS1F and ITS4B [46]; http://www.biology.duke.edu/fungi/mycolab/primers.htm, accessed on 1 March 2017. Sequences of nrLSU-rDNA were generated using primers LR0R and LR5 [47]. Primers EF1-983F and EF1-1567R were used to amplify approximately 500 bp of tef1 [48]. For ITS, PCR was carried out under the following cycling parameters: initial denaturation: 98 °C for 4 min; followed by 35 cycles: 98 °C for 1 min, 52 °C for 1 min, and 72 °C for 1 min, and final extension at 72 °C for 3 min. For nrLSU: initial denaturing at 98 °C for 5 min; then 12 cycles of denaturing at 98 °C for 5 s, annealing at 67 °C for 1 min, extension at 72 °C for 1.5 min; then 35 cycles of denaturing at 98 °C for 5 s, annealing at 56 °C for 1 min, extension at 72 °C for 1.5 min; and a final extension step of 72 °C for 10 min. For *tef1-a*: initial denaturing at 98 °C for 5 min; then 8 cycles of denaturing at 98 °C for 5 s, annealing at 60 °C for 40 s, extension at 72 °C for 2 min; then 36 cycles of denaturing at 98 °C for 5 s, annealing at 53 °C for 1.5 min, extension at 72 °C for 2 min; and a final extension step of 72 °C for 10 min.

PCR products were visualized using agarose gel electrophoresis and Gel Red staining, and subsequently purified with the Fermentas Genomic DNA Purification Kit (Thermo Fisher Scientific Inc., Waltham, MA, USA). Sequencing was performed with an ABI model 3500 Genetic Analyzer (Applied Biosystems, Foster City, CA, USA).

This work was carried out using equipment of the Core Facility Centre ‘Cell and Molecular Technologies in Plant Science’ of the Komarov Botanical Institute. Raw data were edited and assembled in MEGA X [49]. Newly generated sequences have been deposited in the GenBank.

### 2.4. Alignment and Phylogenetic Analyses

For this study, 12 nrITS and 9 *tef1α*, and 10 nrLSU sequences were newly generated. In addition, 38 nrITS and 20 *tef1α*, and 43 nrLSU sequences, including outgroups, were retrieved from the GenBank database (www.ncbi.nlm.nih.gov/genbank, accessed on 15 March 2023), using the BLASTn application (https://blast.ncbi.nlm.nih.gov/Blast.cgi, accessed on 15 March 2023). The information on all these sequences is presented the Table 1.

Three datasets were analysed: nrITS, *tef1α*, and nrLSU. DNA sequences were aligned with the MAFFT v.7.110 web tool [56] using the G-INS-i option, and then manually modified where necessary in MEGA X [49]. To determine the phylogenetic positions of the studied collections, both datasets were analysed using Bayesian Analysis (BA). BA was performed using MrBayes 3.2.1 [57], under a GTR model. The analyses were run with two parallel searches: four chains for 5 million generations for ITS and LSU and for 1 million generations for *tef1α*, starting with a random tree. The trees were sampled every 100 generations. To check for convergence of MCMC analyses and to obtain estimates of the posterior distribution of parameter values, Tracer v1.7.2 was used [58]. The phylogenetic trees were edited in Adobe Illustrator CS4. Posterior probability (PP) values ≥ 0.95 are considered significant.

## 3. Results

### 3.1. Phylogenetic Analysis

The full nrITS dataset contained 50 sequences with 1059 characters (gaps included). The/Entocybe clade was selected as outgroup due to its basal position in the *Entoloma* phylogeny [6]. Besides our specimens, the tree includes 7 more sequences of the subgenus *Cubospora* retrieved from the GenBank NCBI data base, 16 representatives of the subgenus *Leptonia*, and 1–3 representatives of the other main subdivisions of the *Entoloma* s.l.

Since in the GenBank the ITS data are absent for many species of the subgenus *Cubospora,* a tree was constructed for this subgenus based on the *tef1α* as well. For the subgenus *Leptonia*, such information is insufficient for analysis. The full *tef1α* dataset contained 26 sequences with 535 characters (gaps included). It included *Entoloma prunuloides* as an outgroup due to its basal position in the *Entoloma* phylogeny [53,54], and *E. luteolamellatum*, the type-species of the subgenus *Cubospora*. In addition to our specimens, 11 more representatives of this subgenus and 1–3 representatives of the other main subgenera of the *Entoloma* s.l. were included in the analysis.

The full nrLSU dataset contained 52 sequences with 781 characters (gaps included). *Clitopilus prunulus* and *Clitopilopsis hirneola* were chosen as an outgroup because of their basal position in the *Entolomataceae* phylogeny [5]. In addition to new species specimens, 8 representatives of the subgenus *Cubospora* and 15 sequences of the subgenus *Leptonia*, as well as 1–3 sequences of the other main subgenera of the *Entoloma* s.l., were added to the analysis.

**Figure 1:**
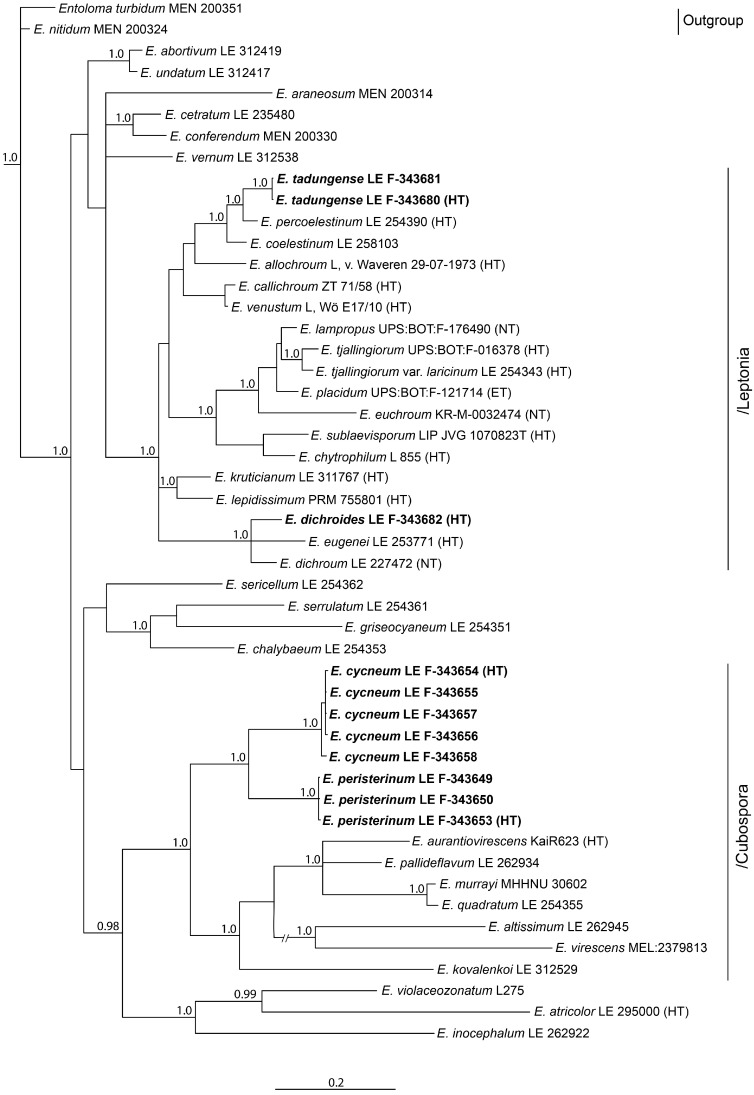
Phylogenetic tree derived from Bayesian analysis, based on nrITS1-5.8S-ITS2 region data. Posterior probability (PP > 0.95) values from the Bayesian analysis are added at the nodes. The scale bar represents the number of nucleotide changes per site. The new species are in bold. HT—holotype.

**Figure 2:**
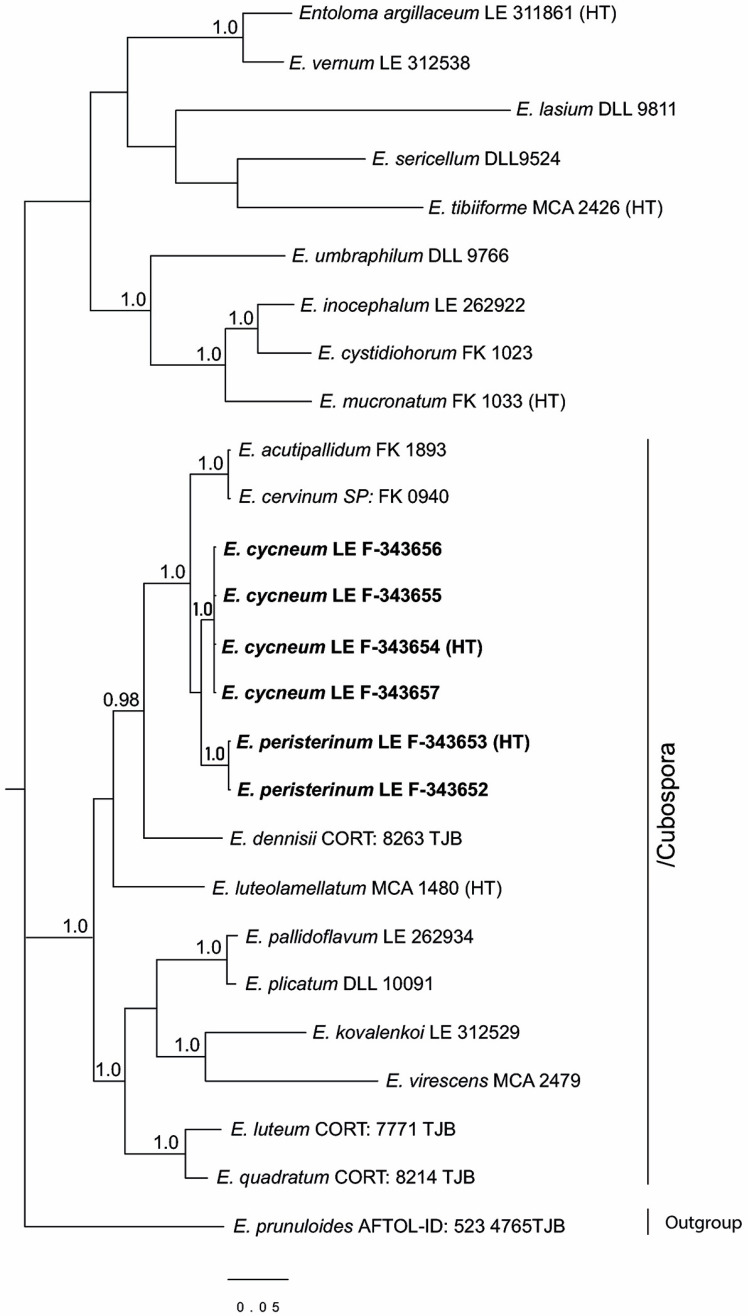
Phylogenetic tree derived from Bayesian analysis, based on *tef1α* data. Posterior probability (PP > 0.95) values from the Bayesian analysis are added at the nodes. The scale bar represents the number of nucleotide changes per site. The new species are in bold. HT—holotype.

**Figure 3:**
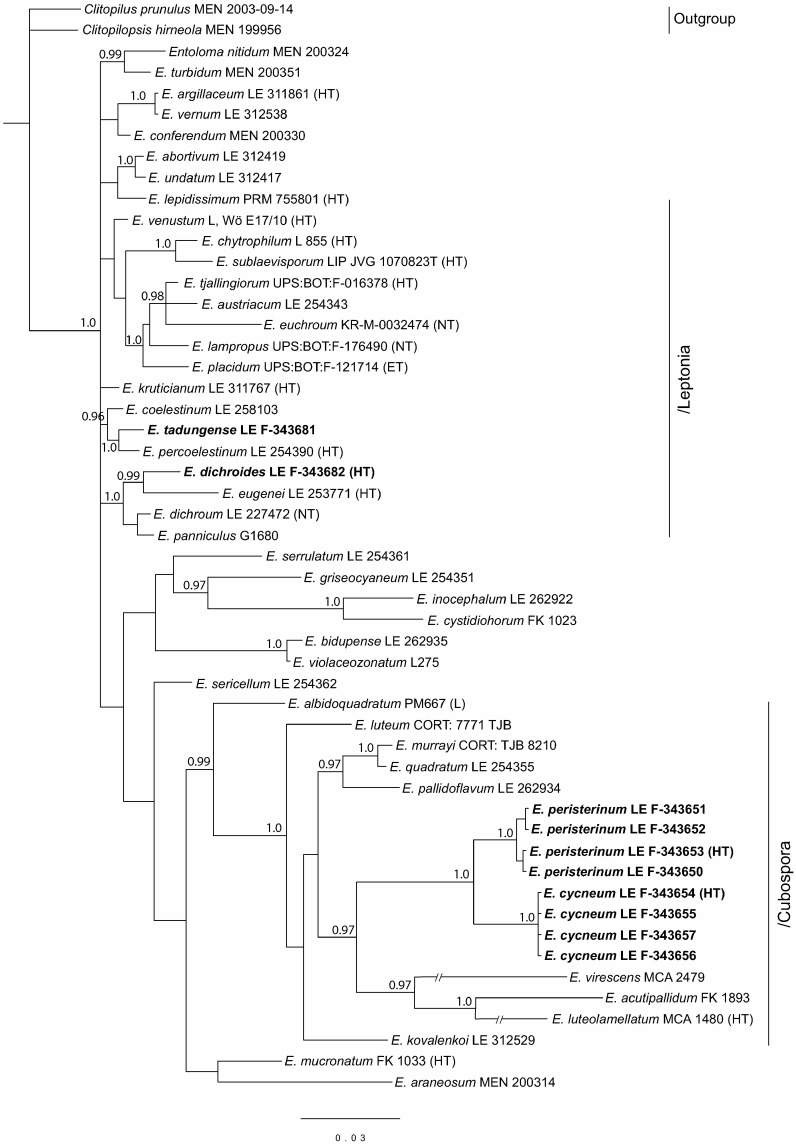
Phylogenetic tree derived from Bayesian analysis, based on nrLSU data. Posterior probability (PP > 0.95) values from the Bayesian analysis are added at the nodes. The scale bar represents the number of nucleotide changes per site. The new species are in bold. HT—holotype.

The results of the phylogenetic analysis are presented in the Figure 1 (nrITS), Figure 2 (*tef1α*), and Figure 3 (nrLSU). *Entoloma cycneum* and *E. peristerinum* form highly supported branches within the/Cubospora clade in all trees. *E. tadungense* and *E. dichroides* clearly nest within the/Leptonia clade in the ITS tree. They are not represented on the *tef1α* tree. Generally, in the LSU tree, the topology mostly is not or hardly resolved due to small differences between the sequences in the subgenera. At the same time, the subgenus *Cubospora* forms a highly supported clade in which two new species (*E. cycneum* and *E. peristerinum*) nest.

Subgenus *Leptonia* does not form a separate clade in the LSU tree, but it is represented by several small clades and singletons. However, it is clearly seen here that *Entoloma tadungense* clusters together with *E. percoelestinum* and *E. coelestinum* with high support. *Entoloma dichroides* distinctly nests in the/dichroum clade, but it differs from the known *Leptonia* species.

### 3.2. Taxonomy

***Entoloma*** subgenus ***Cubospora*** Karstedt, Capelari, Largent, T. J. Baroni & Bergemann, in Phytotaxa 391(1): 20 (2019).

**Figure 4:**
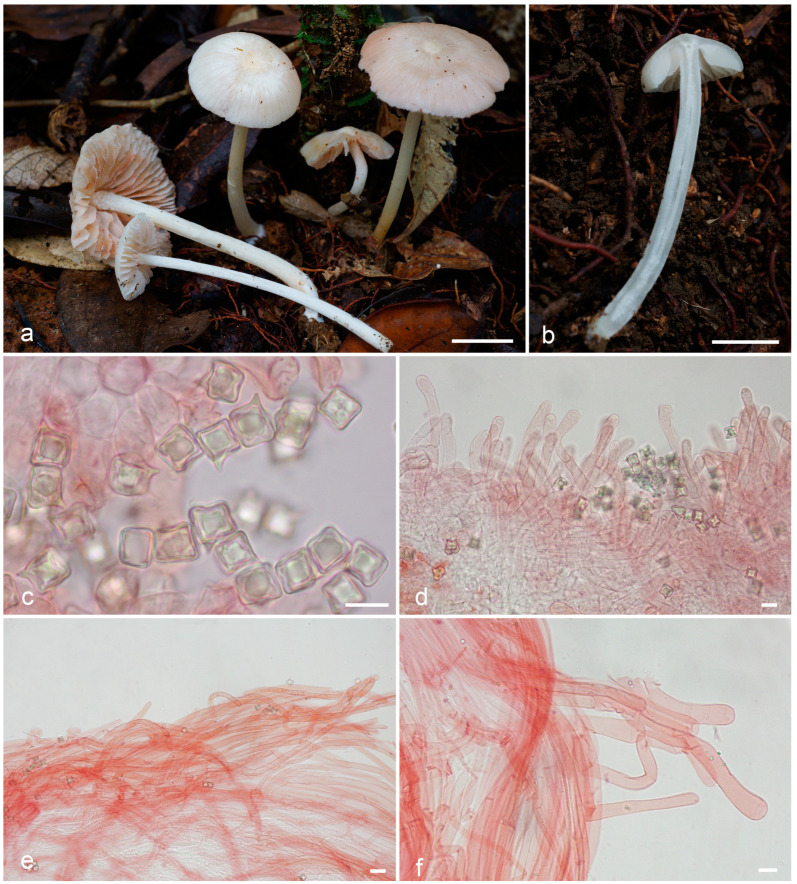
*Entoloma cycneum*: (**a**,**b**). basidiocarps; (**c**). basidiospores; (**d**). cheilocystidia; (**e**). pileipellis; (**f**). caulocystidia (**a**,**d**–**f**, from LE F-343654, holotype; **b**—from LE F-343655). Scale bars (**a**,**b**) 1 cm, (**c**–**f**) 10 μm.

***Entoloma cycneum*** O.V. Morozova & T.H.G. Pham, sp. nov. (Figure 4).

MycoBank: MB848527.

*Etymology*. From “κύκνος” (Greek)—swan, due to colour similarity with white swan.

*Holotype.* Vietnam, Gia Lai Province, K’Bang District, Son Lang Commune, Kon Chu Rang Nature Reserve, N 14.49436°, E 108.54428°, 1030 m a. s. l., on soil in middle-mountain evergreen mixed forest with a predominance of Podocarpaceae (*Dacrydium elatum, Dacrycarpus imbricatus*), Magnoliaceae, Burseraceae (*Canarium*), Myrtaceae (*Syzygium*), 30 May 2016, O.V. Morozova (LE F-343654). Isotype in VRTC (299VN16).

*Diagnosis. Entoloma cycneum* is characterized by initially white or whitish basidiomata with mainly smooth glabrous with thin pubescence near the margin, hygrophanous pileus, longitudinally fibrillose or fibrillose-scaly stipe, cuboid spores, and long, more or less cylindrical cheilocystidia of serrulatum-type.

*Basidiomata* small to medium-sized. *Pileus* 10–25 mm diam., firstly hemispherical, then convex, plano-convex with small papilla, with a slightly involute then straight crenulate margin, hygrophanous, smooth, mostly glabrous, but covered with thin fibrils on the pileus margin, in wet conditions translucently striate almost up to the centre, white to cream (3A1–2; 4A1–2), yellowish in places of damage, lighter towards margin, slightly darker in radial hygrophanous stripes, then dries up to white. *Lamellae* moderately distant, adnexed, adnate-emarginate, ventricose, initially white, then cream, pale pink, with white denticulate edge. *Stipe* 30–60 × 1.5–3 mm, cylindrical, fistulous, white, longitudinally fibrillose or fibrillose-scaly, at least at the top, white tomentose at base. Context white or hyaline. Smell indistinct, taste not reported.

*Basidiospores* (7.2–)8.5–9.0(–9.7) × (7.2–)8.0–8.5(–8.7) μm, Q = 1.0–1.2, isodiametrical or subisodiametrical, cuboid, rarely with 5 angles in side-view. *Basidia* 37–50 × 11–12.5 μm, 4-spored, clavate, constricted in the middle part, clamped. *Cheilocystidia* 95–160 × 7.5–9 μm, of serrulatum-type, flexuous-cylindrical, sometimes capitate or narrowly clavate, septate, not pigmented, with granular content, forming sterile lamellae edge. *Hymenophoral trama* regular, made up of cylindrical or inflated hyphae 4–10 μm wide. *Pileipellis* a cutis of cylindrical hyphae up to 10 μm broad, some hyphae are rising. *Pileitrama* consists of cylindrical hyphae 4–6 μm broad, intertwined by oleiferous hyphae with brilliant content. *Stipitipellis* of cylindrical hyphae up to 7 μm broad. *Caulocystidia* in bundles of rising hairs, cylindrical or slightly broadened towards apex, up to 250 μm length and 12 μm broad. Clamp-connections present in all tissue. *Brilliant* granules abundant.

*Habitat and distribution*—In small groups on soil in middle-mountain evergreen mixed forest. Known from Vietnam.

*Additional specimens examined*. Vietnam, Gia Lai Province, K’Bang District, Son Lang Commune, Kon Chu Rang Nature Reserve, N 14.49439°, E 108.54591°, 990 m a. s. l., on soil in middle-mountain evergreen mixed forest with a predominance of Podocarpaceae (*Dacrydium elatum, Dacrycarpus imbricatus*), Magnoliaceae, Burseraceae (*Canarium*), Myrtaceae (*Syzygium*), 27 May 2016, O.V. Morozova (LE F-343656 (1687_243VN16); ibid., 28 May 2016, O.V. Morozova (LE F-343655 (1689_255VN16); LE F-343657 (1690_256VN16); Dak Nong Province, Dak Plao District, south-east macroslope of Mt. Ta Dung, trail along the stream valley in the saddle between M’neun Tchirke and the eastern spur of Ta Dung, N 11.87172°, E 108.08253°, 1310 m a. s. l., on soil in middle-mountain evergreen mixed forest with a participation of Fagaceae and Lauraceae (*Litsea glutinosa*), 3 June 2022, T.H.G. Pham (LE F-343658).

**Figure 5:**
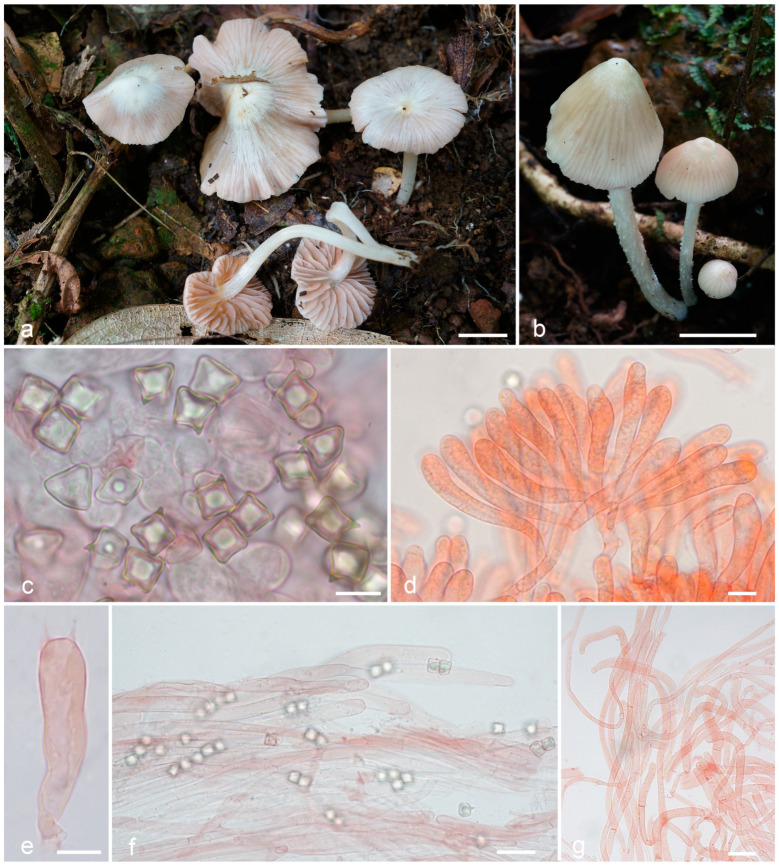
*Entoloma peristerinum*: (**a**,**b**). basidiocarps; (**c**). basidiospores; (**d**). cheilocystidia; (**e**). basidium; (**f**). pileipellis; (**g**). caulocystidia (**a**,**c**–**g**, from LE F-343653, holotype; **b**—from LE F-343652). Scale bars (**a**,**b**) 1 cm, (**c**–**e**) 10 μm, (**f**,**g**) 20 μm.

***Entoloma peristerinum*** O.V. Morozova & T.H.G. Pham, sp. nov. (Figure 5).

MycoBank: MB848528.

*Etymology*. From “περιστερά” (Greek)—dove, due to colour similarity to white dove.

*Holotype.* Vietnam, Gia Lai Province, K’Bang District, Son Lang Commune, Kon Chu Rang Nature Reserve, N 14.49667°, E 108.56106°, 980 m a. s. l., on soil in middle-mountain evergreen mixed forest with a predominance of Podocarpaceae (*Dacrydium elatum*, *Dacrycarpus imbricatus*), Magnoliaceae, Burseraceae (*Canarium*), Myrtaceae (*Syzygium*), 28 May 2016, O.V. Morozova (LE F-343653). Isotype in VRTC (276VN16).

*Diagnosis. Entoloma peristerinum* is characterized by initially cream, pale beige to beige or greyish-beige becoming white basidiomata, with smooth, glabrous, hygrophanous pileus, longitudinally fibrillose or fibrillose-scaly stipe, cuboid spores and cheilocystidia mostly narrowly clavate or tapering towards the apex—of porphyrogriseum-type.

*Basidiomata* small to medium-sized. *Pileus* 10–30 mm diam., firstly conical, then broadly conical with distinct acute papilla, with deflexed then straight or undulating, slightly crenulate margin, hygrophanous, smooth, glabrous, in wet condition translucently striate almost up to the centre, cream, pale beige to beige or greyish-beige (4A2; 4B2–3; 5B2–3), lighter towards margin, with dark radial hygrophanous stripes and yellowish papilla, then dries up by white radial strokes, finally becomes completely white, silky. *Lamellae* moderately distant, adnate-emarginate, ventricose, cream, pale pink, with minutely serrate whitish edge. *Stipe* 30–70 × 1.5–3 mm, cylindrical, fistulous, white, longitudinally fibrillose or fibrillose-scaly, at least at the top, white tomentose at base. Context white. Smell indistinct, taste not reported.

*Basidiospores* (7.4–)8–9.5(–9.8) × (6.5–)7–8(–9.2) μm, Q = 1.0–1.2(–1.3), isodiametrical or subisidiametrical, cuboid, rarely with 5 angles in side-view. *Basidia* 47–60 × 11–14.5 μm, 4-spored, clavate, constricted in the middle part, clamped. *Cheilocystidia* 75–215 × 12–15 μm, of porphyrogriseum-type [8], cylindrical, narrowly clavate or tapering towards the apex, with granulose content, usually forming sterile lamellae edge; in young basidiomata lamellae edge can be heterogeneous with rare cheilocystidia. *Hymenophoral trama* regular, made up of cylindrical or inflated hyphae 3–8 μm wide. *Pileipellis* a cutis of cylindrical hyphae up to 10 μm broad. *Pileitrama* consists of cylindrical hyphae 4–6 μm broad, intertwined by oleiferous hyphae with brilliant content. Clamp-connections present in all tissue. Brilliant granules abundant.

*Habitat and distribution*—In small groups on soil in middle-mountain evergreen mixed forest. Known from Vietnam.

*Additional specimens examined*. Vietnam, Gia Lai Province, K’Bang District, Son Lang Commune, Kon Chu Rang Nature Reserve, N 14.49439°, E 108.54591°, 990 m a. s. l., on soil in middle-mountain evergreen mixed forest with a predominance of Podocarpaceae (*Dacrydium elatum, Dacrycarpus imbricatus*), Magnoliaceae, Burseraceae (*Canarium*), Myrtaceae (*Syzygium*), 28 May 2016, O.V. Morozova (LE F-343651 (1688_254VN16)); ibid., N 14.49667°, E 108.56106°, 980 m a. s. l., 28 May 2016, O.V. Morozova (LE F-343650 (1691_276bVN16)); ibid., N 14.49436°, E 108.54428°, 1030 m a. s. l., 30 May 2016, O.V. Morozova, LE F-343652 (1692_300VN16), LE F-343649 (1696_312VN16)).

Notes—*Entoloma cycneum* and *E. peristerinum* are morphologically very similar species characterized by white or whitish with yellowish or beige tinged basidiomata with mainly smooth, glabrous, and hygrophanous pileus, longitudinally fibrillose or fibrillose-scaly white stipe, cuboid spores and more or less cylindrical cheilocystidia, arising from hymenophoral trama. *Entoloma peristerinum* posseses initially more coloured beige conical pileus, discolouring to white with age and drying. The pileus of *E. cycneum* is initially white, hemisphaerical to convex, usually with thin pubescence near the margin. *E. peristerinum* differs from *E. cycneum* also by more differentiated porphyrogriseum-type [8] cheilocystidia, vs. serrulatum-type in *E. cycneum*. Yellow tinge can present in old or damaged basidiomata of both species.

Phylogenetically both new species are also close to each other but rather distant from other known species in the subgenus *Cubospora* [7]. *Entoloma cervinum* (Karstedt & Capelari) Blanco-Dios and *E. acutipallidum* E. Horak & Cheype from the South America are the closest species according to the phylogenetical analysis. Morphologically they differ from our species by darker, distinctly coloured pileus [28].

Several species with cuboid spores and predominantly white or whitish basidiomata have been described at different times from different regions of the Earth.

The pileus of *Entoloma alboumbonatum* Hesler from North America is darker coloured, and only umbo is white. This species is characterized by the clavate or capitate cheilocystidia, presence of the pleurocystidia, and absence of the clamp connections [31].

*E. albidoquadratum* Manim. & Noordel., described from India, is characterized by non hygrophaneous, non-translucently striate pileus, presence of pleurocystidia, and large spores [20]. *E. minutoalbum* E. Horak is a species of Southern Hemisphere, a common fungus of the subantarctic *Nothofagus* forests of Tierra del Fuego and New Zealand. Morphologically it differs from new species by small basidiomata (with the pileus less than 10 mm diam.), smaller spores, and absence of the cheilocystidia [13]. *E. albogracile* E. Horak is also species from the Southern Hemisphere—Papua New Guinea. It is characterized by the small size, pileus covered with minute scales and fibrils, and by the absence of clamp-connections [13]. *E. laccaroides* T.H. Li, E. Horak & Xiao Lan He is recognized by the umbilicate pileus, and numerous conspicuous broadly fusoid to utriform pleurocystidia [12].

Due to the discolouration new species would be compared with cuboid-spored light-yellow species. *Entoloma dennisii* from Trinidad is a rather robust species with pileus up to 5 cm broad, deep to pale yellow colour, and small spores. *E. pallidoflavum* differs by the predominance of light-yellow colour in the pileus, yellow content of the oleiferous hyphae, and smaller spores [13].

***Entoloma* subgen. *Leptonia* (Fr.) Noordel.**, in Persoonia 11: 146 (1981), emend. O. V. Morozova, Noordel. & Vila (2014); emend. Noordeloos et al. (2022).

**Figure 6:**
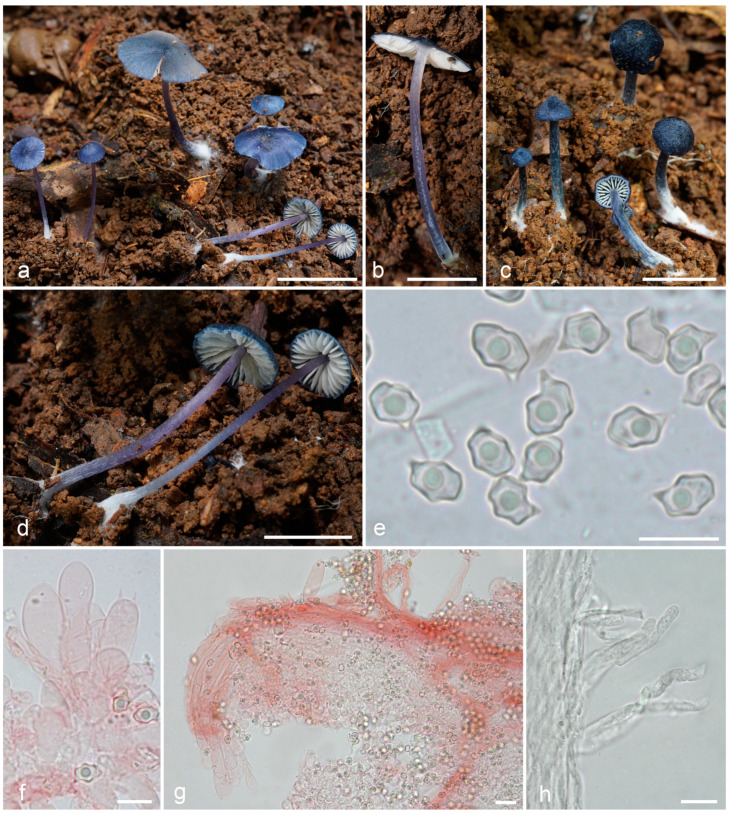
*Entoloma tadungense*: (**a**–**d**). basidiocarps; (**e**). basidiospores; (**f**). cheilocystidia and basidium; (**g**). pileipellis; (**h**). caulocystidia (**a**–**f**,**h** from LE F-343680, holotype; **g**—from LE F-343681). Scale bars (**a**–**d**)—1 cm; (**e**,**f**,**h**)—10 μm; (**g**)—20 μm.

***Entoloma tadungense*** O.V. Morozova & T.H.G. Pham, sp. nov. (Figure 6).

MycoBank: MB848530.

*Etymology*. Named after the type-locality—Ta Dung National Park.

*Holotype*. Dak Nong Province, Dak Glong District, Ta Dung National Park, south-eastern macroslope of the ridge of the Ta Dung Mt, south-eastern slope of the Ta Dung Mt, TK 1805, N 11.86780°, E 108.11692°, 1240 m a.s.l., on soil in evergreen broad-leaved forest with *Lithocarpus* spp., *Quercus* sp., *Schima* sp., *Acer flabellatum*, Dilleniaceae, Myristicaceae, 11 October 2022, T.H.G. Pham, O.V. Morozova (LE F-343680). Isotype in VRTC (87VN22).

*Diagnosis*. *Entoloma tadungense* is distinguished among the other *Leptonia* species by the tiny dark blue basidiomata with discolouring to lilac of the stipe, and by the small spores with rather pronounced angles.

*Basidiomata* small-sized, mycenoid. Pileus 3–10 mm broad, conical, or hemispherical with umbo, becoming almost applanate, not hygrophanous, not translucently striate, with deflexed then straight margin, radially fibrillose, squamulose at centre, firstly uniformly dark blue, blackish blue (19F7–8; 20E6–8; 21F7–8), glabrescent, radially cracking showing the white background, and pallescent (up to 20C5–6; D5–6) with edge. *Lamellae* moderately distant, adnate emarginate, ventricose, white, becoming pinkish, with entire concolourous edge. *Stipe* 1.5–30 × 0.5–2 mm, cylindrical or broadened towards to base, fistulous, initially distinctly longitudinally fibrillose, glabrescent with age up to almost smooth, especially in the apex, firstly concolourous with pileus—dark blue, blackish blue (19F7–8; 20E6–8), then discoloured up to lilac, whitely tomentose at base. Context thin, concolourous with the surface. *Smell* indistinct, taste not reported.

*Basidiospores* (6.0–)6.8–6.9(–8.3) × (4.2–)4.8–4.9(–5.7) μm, Q = 1.3–1.4(–1.6), heterodiametrical, with 6–7 relatively sharp angles in side-view. *Basidia* 19.5–24 × 6.6–8.3 μm, 4-spored, narrowly clavate to subcylindrical, clamped. *Lamellae edge* fertile or heterogeneous. *Cheilocystidia* 24–67 × 9–17 μm, of poliopus-type [8], clavate or lageniform, more frequent near the pileus margin. *Hymenophoral trama* regular, made up of narrow, cylindrical hyphae up to 3 μm wide. *Pileipellis* a cutis with a transition to a plagiotrichoderm and a trichoderm of cylindrical to slightly inflated hyphae 10–20 μm wide with blue intracellular pigment. *Stipitipellis* a cutis with a transition to a plagiotrichoderm with rising hyphae forming hairs up to 5 μm wide. *Clamp-connections* present, but rare. Brilliant granules absent.

*Habitat and distribution*—In small groups on soil in middle-mountain evergreen mixed forest. Known from Vietnam.

*Additional specimens examined*. Dak Nong Province, Dak Glong District, Ta Dung National Park, south-eastern macroslope of the ridge of the Ta Dung Mt, south-eastern slope of the Ta Dung Mt, TK 1805, N11.86780, E 108.11692, 1240 m a.s.l., on soil in evergreen broad-leaved forest with *Lithocarpus* spp., *Quercus* sp., *Schima* sp., Dilleniaceae, Myristicaceae, *Acer flabellatum*, 11 Oct. 2022, T.H.G. Pham, O.V. Morozova, LE F-343681, duplicate in VRTC (88VN22); ibid., 15 October 2022, LE F-343683, duplicate in VRTC (138VN22), LE F-343684, duplicate in VRTC (139VN22).

Notes—*Entoloma tadungense* is a species of the subgenus *Leptonia* due to presence of clamp connections, absence of brilliant granules, longitudinally fibrillose stipe surface and plagiotrichoderm to trichoderm pileipellis. It resembles *Entoloma percoelestinum* O.V. Morozova, Noordel., Vila & Bulyonk. by the small-sized dark blue mycenoid basidiomata [39]. Microscopically the absence of cheilocystidia and small spores also make them similar. However, *E. tadungense* can be recognized by smaller spores with pronounced angles, as well as the lilac discolouration of the stipe. Molecular data support their differences (p-distance from the closest species *E. percoelestinum* (ITS1-5.8S-ITS2 region)—4.9%). The similar non tropical species with dark blue colour and small size of the basidiomata distinguish from the new species in the following: *E. coelestinum* (Fr.) Hesler—by the smooth stipe and slightly larger spores, *E. lepidissimum* (Svrček) Noordel. and *E. venustum*—by the coloured lamellae, distinctly larger spores, and presence of the cheilocystidia, *E. chytrophilum* possesses large nodulose spores [39]. The American species *E. subcoelestinum* (Largent) Blanco-Dios is characterized by the initially coloured lamellae, moniliform cells in the pileipellis, and larger indistinctly angular spores [32].

**Figure 7:**
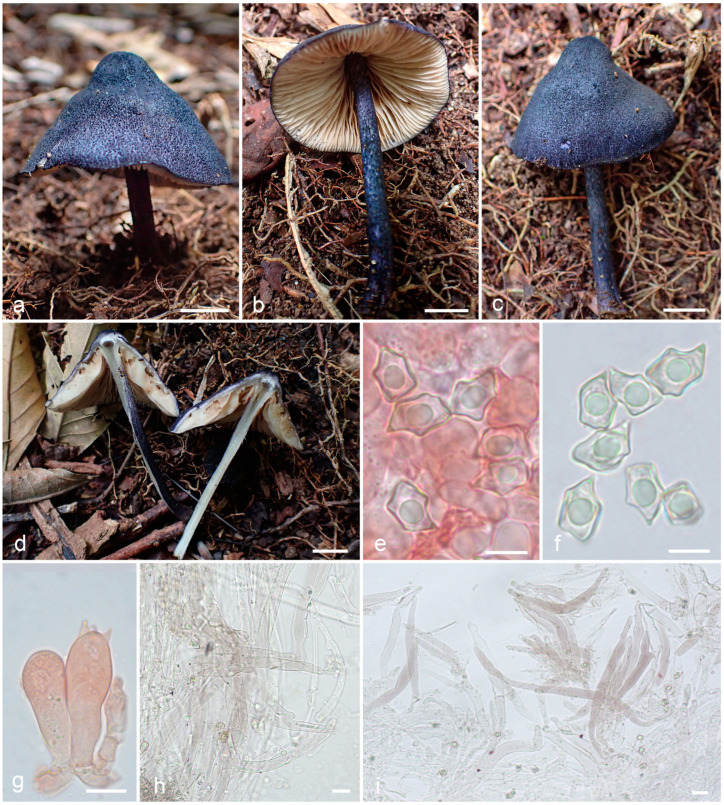
*Entoloma dichroides*: (**a**–**d**). basidiocarps; (**e**,**f**). basidiospores; (**g**). basidiola and basidium; (**h**). stipitipellis; (**i**). pileipellis (all from LE F-343682). Scale bars (**a**–**d**)—1 cm; (**e**–**i**)—10 μm.

***Entoloma dichroides*** O.V. Morozova & T.H.G. Pham, sp. nov. (Figure 7).

MycoBank MB 848531.

*Etymology*. Named after its similarity to *Entoloma dichroum*.

*Holotype*. Dak Nong Province, Dak Glong District, Ta Dung National Park, northwest of the Ta Dung Mt, TK 1781, N11.923056°, E 108.00194°, 1000 m a.s.l., on soil in evergreen broad-leaved forest with *Parashorea chinensis, Rhodoleia championii, Fagaceae, Lauraceae, Hypericaceae*, 1 June 2022, Pham T.H.G., LE F-343682. Isotype in VRTC (HG09).

*Diagnosis*. *Entoloma dichroides* is a species of subgenus *Leptonia*, characterized by the dark blue basidiomata with squamulose surface of the pileus and stipe, initially white lamellae, absence of the cheilocystidia, and spores with 5(–6) pronounced angles and elongated apiculus.

*Basidiomata* medium-sized, mycenoid. *Pileus* 40 mm diam, conical to conico-convex, with deflexed then straight margin, not hygrophanous, not translucently striate, dark blue (20F6–8), entirely fibrillose-squamulose with prominent dense squamules on whitish background. *Lamellae* narrowly adnexed to almost free, ventricose, moderately distant, initially white becoming yellowish-pink, brownish-pink when, with an entire concolourous edge. *Stipe* 60 × 3–4 mm, cylindrical, slightly broadened at base, hollow, dark blue, concolourous with the pileus, entirely fibrillose-squamulose with dark blue fibrils (20F6–8) on whitish background. *Context* white. *Smell* weak, taste not reported. *Basidiospores* 9.5–12.5 × 6.5–8.5 μm, on average 10.8 × 7.2 μm, Q = 1.4–1.6(1.7), Qav = 1.5, 5(–6)-angled, heterodiametric with pronounced angles and elongated apiculus. *Basidia* 31–41 × 10–13 μm, 4-spored, clamped. *Lamella edge* fertile. *Cheilocystidia* absent. *Hymenophoral trama* regular, made up of narrow cylindrical hyphae 2–6 μm wide. *Pileipellis* a trichoderm of cylindrical entangled 6–10 m wide hyphae with fusiform or irregularly shaped terminal elements, 90–200 × 13–19 μm, with dark blue intracellular pigment. *Stipitipellis* an entangled trichoderm of cylindrical hyphae, 6–12 m wide with cylindrical to lageniform, terminal elements, forming squamules, 4–12 μm wide and up to the 200 long, with dark blue intracellular pigment. *Clamp-connections* abundant. *Brilliant granules* absent.

*Habitat and distribution*—Solitary in middle-mountain evergreen mixed forest. Known from Vietnam.

Notes—*Entoloma dichroides* is similar to European *E. dichroum* and Australian *E. panniculus* due to dark blue basidiomata with squamulose surface of the pileus and stipe, initially white lamellae, and spores with pronounced angles [26]. New species differs from them by darker basidiomata with conical pileus, basidiospores with elongated apiculus, and absence of the cheilocystidia. The Eastern species *E. eugenei* Noordel. & O.V. Morozova is also close. It is recognized by the more robust basidiomata and presence of the cheilocystidia [21]. The p-distance (ITS1-5.8S-ITS2 region) of the new species from *E. dichroum*—6.9%, from *E. eugenei*—10.4%.

## 4. Discussion

The genus *Entoloma* is the second largest genus in the order Agaricales with over 1000 species worldwide [59]. However, studies devoted to it in Vietnam have not yet been carried out enough.

The first most important contribution to the study of Vietnamese mycobiota was made by N. Patouillard, who described many new species from Northern and Central Vietnam based on collections by V. Demange, L. Duport, P. A. Eberhardt, E. Poilane, and others. First records of the genus *Entoloma* (*Rhodophyllus clypeatus*, *Rh. sericeus*) were also published by him [60,61], including originally described from Vietnam *Rh. submurinus* [61]. Heim and Malençon [62] published information on *E. madidum*. The first checklist summarizing all the data on the species composition of fungi and slime-molds in Vietnam was published in 1998 and included 829 species, of which only 6 belonged to the genus *Entoloma* [63]. The same species are presented in the list of plant species of Vietnam [64]. In 2003, Le Ba Dung listed 300 species of macromycetes for the Central Highlands, with only two *Entoloma* species: *E. lividum* and *E. madidum* [65]. Later, Ngô Anh and Nguyễn Thị Kim Cúc recorded two more species of the genus in Thua Thien Hue Province—*E. prunuloides* in Bach Ma National Park [66] and *E. abortivum* in Phong Dien Nature Reserve [67]. The data on Vietnamese mycobiota have been summarized in a monography published in 2011–2012, which provides information on the ecology and distribution of more than 900 species of macromycetes [68,69] with only 3 *Entoloma* species.

More intensive studies of the genus *Entoloma* in Vietnam began in the frame of the work of the Joint Vietnam–Russia Tropical Science and Technology Research Centre in collaboration with the Komarov Botanical Institute RAS. In 2012, Morozova et al. [11] reported 12 species of the genus *Entoloma* (Agaricales, Basidiomycota) for the Bidoup—Nui Ba (Lam Dong Province) and Cat Tien (Dong Nai Province) National Parks, of which 11 species were recorded for Vietnam for the first time, and a new species for science, *E. myriadophyllum* O.V. Morozova, was described.

The above information was based only on morphological data. In connection with the revision of the system of the genus *Entoloma* [8], these data are not entirely credible and require confirmation. In cases where herbarium material is absent or old and destroyed, this is not possible. However, sequences were later obtained for some of these records. Some of the identifications were revised, and some of these findings were described as species new to science—*E. daphnis* and *E. bidupense* [4,42]. Our specimens with cuboid spores were used in the work of Karstedt and colleagues [7], where additional data on *tef1α*, mtSSU, rpb2, and LSU markers were obtained for them.

Further descriptions of new species have already been confirmed by molecular data. Thus, *Entoloma flavovelutinum* and *E. porphyroleucum* from the Bu Gia Map National Park (Binh Phuoc Province) [2,70], *E. nigrovelutinum* from Chu Yang Sin National Park (Dak Lak Province) [71], and *E. atricolor* [72], *E. arion*, *E. argus*, *E. daphnis*, *E. icarus* [4], *E. kovalenkoi* [3], from the Kon Chu Rang Nature Reserve were described.

As a result, the data on 29 species of *Entoloma* have been published for Vietnam so far. Only for 14 of them the molecular data have been published and submitted into the GenBank. The resulting list is presented here.

List of the species of the genus *Entoloma* mentioned in the publications for Vietnam, 1910–2022 (species, supported for the molecular data are marked by the asterisk (*)):

*Entoloma abortivum* (Berk. & M.A. Curtis) Donk [67]

*E. altissimum (Massee) E. Horak [11]

*E. argus O.V. Morozova, E.S. Popov, A.V. Alexandrova & Noordel. [4]

*E. arion O.V. Morozova, E.S. Popov, T.H.G. Pham & Noordel. [4]

*E. atricolor O.V. Morozova, Noordel., E.S. Popov & A.V. Alexandrova [42,72]

*E. bidupense O.V. Morozova & E.S. Popov [11] (as *E. violaceoserrulatum* Noordel.), [42]

*E. carneum Z.S. Bi [7,11]

*E. chalybeum* (Fr.) Noordel. var. *lazulinum* (Fr.) Noordel. [11], (non est)

*E. clypeatum* (L.) P. Kumm. [60] (as *Rh. clypeatus* Fr.), [63,64,68]

*E. daphnis O.V. Morozova, E.S. Popov, T.H.G. Pham & Noordel. [4,11] (as *E. nubilum* Manim., Leelav. et Noordel.)

*E. depluens* (Batsch) Hesler [63,64]

*E. flavovelutinum O.V. Morozova, E.S. Popov, A.V. Alexandrova & Xiao L. He [70]

*E. icarus O.V. Morozova, E.S. Popov & Noordel. [4]

*E. inocephalum (Romagn.) Dennis [7,11]

*E. kovalenkoi O.V. Morozova, E.S. Popov & A.V. Alexandrova [3]

*E. sinuatum* (Bull.) P. Kumm. [65,68] (both as *E. lividum* (Bull.) Quél.)

*E. longistriatum* (Peck) Noordel. [11]

*E. madidum* Gillet [62] (as *Rhodophyllus madidus* (Fr.) Quél.), [63,64,65]

*E. myriadophyllum O.V. Morozova [11]

*E. nigrovelutinum O.V. Morozova & A.V. Alexandrova [71]

*E. pallidoflavum (Henn. & E. Nyman) E. Horak [7,11]

*E.* cf. *platyphylloides* (Romagn.) Largent [73]

*E. poliopus* (Romagn.) Noordel. var. *alpigenum* (J. Favre) Bon [11], (non est)

*E. porphyroleucum O.V. Morozova, Noordel. & Dima [2]

*E. prunuloides* (Fr.) Quél. [63,64,66]

*E. quadratum (Berk. et M. A. Curtis) E. Horak [11]

*E. sericeum* (Bull.) Quél. [61] (as *Rh. sericeus* (Fr.) Quél.), [63,64]

*E. submurinum* (Pat.) E. Horak [61] (as *Rh. submurinus* Pat.), [63,64]

*E. sulcatum* (T.J. Baroni et Lodge) Noordel. et Co-David [11]

Four more species are described in the presented article. For *E. pallidoflavum*, the information on ITS sequence is published here.

In conclusion, it is important to note that the area of tropical forests has been drastically reduced in recent years, including in Vietnam. Along with them, the species confined to them disappear, often without even being assigned a name. Nature reserves and national parks serve to save biologically valuable forest areas. However, even here the species are vulnerable. It sometimes happens that type localities are destroyed during road reconstructions (e.g., *E. atricolor*, *E. arion*). It remains to be hoped that they will continue to be found in the adjacent forests. In central Vietnam, the greatest diversity of fungi of the genus *Entoloma* was observed in middle-mountain evergreen broad-leaved forests. They require special attention, study, and careful treatment.

The study of the fungal diversity of typical tropical forests complements the information on the biota of macromycetes in Vietnam and may be useful in the development of measures for the conservation of these valuable nature areas and the species inhabiting them.

## Figures and Tables

**Table 1:** Specimens and GenBank accession numbers of DNA sequences used in the molecular analyses (newly generated sequences are in bold).

*Species*	Location	Voucher Number	Genbank Accession No.			References
			ITS	LSU	tef	
*Clitopilopsis hirneola* (Fr.) Kühner (*Clitopilus hirneolus* (Fr.) Kühner & Romagn.)	Italy	MEN 199956	—	GQ289211	—	[5]
*Clitopilus prunulus* (Scop.) P. Kumm.	Belgium	MEN 2003-09-14	—	GQ289149	—	[5]
*Entoloma abortivum* (Berk. & M.A. Curtis) Donk	Russia: Far East	LE 312419	MF476905	MF487792	—	[42]
*E. acutipallidum* E. Horak & Cheype	Brazil: Pará	SP: FK1893	—	MG018325	MH190147	[7]
*E. albidoquadratum* Manim. & Noordel.	India: Kerala	PM667 (L)	—	GQ289151	—	[5]
*E. allochroum* Noordel.	The Netherlands	v. Waveren, 29-07-1973, holotype (L)	KC898372	—	—	[39]
*E. altissimum* (Massee) E. Horak	Vietnam	LE 262945	MF476912	—	—	[42]
*E. argillaceum* O.V. Morozova et al.	Russia: Caucasus	LE 311861, holotype	—	OL338531	OL405537	[38]
*E. araneosum* (Quél.) M.M. Moser	Belgium	MEN 200314	KC710056	GQ289153	—	[5]
*E. atricolor* O.V. Morozova et al.	Vietnam	LE 295000, holotype	KY777496	—	—	[42]
*E. aurantiovirescens* Reschke, Lotz-Winter & Noordel.	Panama	KaiR623, holotype	MZ611665	—	—	[50]
*E. austriacum* Courtec. [*E. tjallingiorum* var. *laricinum* O.V. Morozova et al.]	Russia: Far East	LE 254343, holotype	KC898413	KC898513	—	[39]
*E. bidupense* O.V. Morozova & E.S. Popov	Vietnam	LE 262935	—	NG_059265	—	[42]
*E. callichroum* E. Horak & Noordel.	Switzerland	ZT 71/58, holotype	KC898350	—	—	[39]
*E. cervinum* (Karstedt & Capelari) Blanco-Dios [*Inocephalus cervinus* Karstedt & Capelari]	Brazil	SP: FK 0940	—	—	MH190138	[7]
*E. cetratum* (Fr.) M.M. Moser	Russia: European part	LE 235480	KC898450	—	—	[39]
*E. chalybaeum* (Pers.) Noordel.	Russia: European part	LE 254353	KC898445	KC898500	—	[39]
*E. chytrophilum* Wölfel, Noordel. & Dähncke	Spain: Canary Islands	L 855, holotype	KC898434	KC898519	—	[39]
*E. coelestinum* (Fr.) Hesler	Russia: Ural	LE 258103	KC898362	KC898524	—	[39]
*E. conferendum* (Britzelm.) Noordel.		MEN 200330	KC710055	KC710133	—	[51]
***E. cycneum* O.V. Morozova et T.H.G. Pham**	**Vietnam**	**LE F-343654, holotype**	**OQ779461**	**OQ804518**	**OQ779183**	this work
***E. cycneum***	**Vietnam**	**LE F-343655**	**OQ779463**	**OQ804519**	**OQ779182**	this work
***E. cycneum***	**Vietnam**	**LE F-343656**	**OQ779462**	**OQ804521**	**OQ779181**	this work
***E. cycneum***	**Vietnam**	**LE F-343657**	**OQ779464**	**OQ804520**	**OQ779184**	this work
***E. cycneum***	**Vietnam**	**LE F-343658**	**OQ779465**	—	**OQ779185**	this work
*E. cystidiophorum* Dennis [*Inocephalus cystidiophorus* (Dennis) Karstedt & Capelari]	Brazil: São Paulo	SP: FK1023	—	MG018319	MH190140	[7]
*E. dennisii* E. Horak [*Inocephalus dennisii* (E. Horak) Karstedt & Capelari]	Puerto Rico	CORT: 8263 TJB	—	—	MH190164	[7]
***E. dichroides* O.V. Morozova et T.H.G. Pham**	**Vietnam**	**LE F-343682**	**OQ779472**	**OQ804527**	—	this work
*E. dichroum* (Pers.) P. Kumm.	Russia: European part	LE 227472, neotype	KC898440	—	—	[39]
*E. dichroum*	Russia: European part	LE 234260	—	KC898527	—	[39]
*E. euchroum* (Pers.) Donk	Russia: Caucasus	LE 262995	KC898417	KC898516	—	[39]
*E. eugenei* Noordel. & O.V. Morozova	Russia: Primorsky Territory	LE 253771, holotype	KC898438	KC898529	—	[39]
*E. griseocyaneum* (Fr.) P. Kumm.	Russia: Caucasus	LE 254351	KC898444	KC898498	—	[39]
*E. inocephalum* (Romagn.) Dennis	Vietnam	LE 262922	KC898449	MH259311	MH190154	[7,39]
*E. kovalenkoi* O.V. Morozova, E.S. Popov & A.V. Alexandrova	Vietnam	LE 312529	OK257210	OK257207	OK256169	[3]
*E. kruticianum* O.V. Morozova, M.Y. Dyakov, E.S. Popov & A.V. Alexandrova	Russia: European part	LE 311767, holotype	KU666558	KU710222	—	[52]
*E. lampropus* (Fr.) Hesler	Sweden	UPS:BOT:F-176490, neotype	KC898377	KC898506	—	[39]
*E. lasium* (Berk. & Broome) Noordel. & Co-David [*Pouzarella lasia* (Berk. & Broome) Largent & Abell-Davis]	Australia: Queensland	DLL9811 (BRI, CNS)	—	—	MG702641	[7]
*E. lepidissimum* (Svrček) Noordel.	Czech Republic	PRM 755801, holotype	KC898364	KC898532	—	[39]
*E. luteolamellatum* (Largent & Aime) Blanco-Dios [*Trichopilus luteolamellatus* Largent & Aime]	Guyana	MCA 1480, holotype	—	MH190213	MG702644	[7]
*E. luteum* Peck [*Inocephalus luteus* (Peck) T.J. Baroni]	USA: New York	CORT: 7771 TJB	—	MH190212	MH190161	[7]
*E. mucronatum* (Karstedt & Capelari) Blanco-Dios [*Inocephalus mucronatus* Karstedt & Capelari]	Brazil: São Paulo	SP: FK1033, holotype	—	MH190174	MH190141	[7]
*E. murrayi* (Berk. & M.A. Curtis) Sacc. & P. Syd.	China	MHHNU 30602	MK250917	—	—	[53]
*E. murrayi*	USA	CORT: TJB 8210	—	MH190193	—	[7]
*E. nitidum* Quél.	Slovakia	MEN 200324	KC710122	GQ289175	—	[5]
***E. pallidoflavum*** **(Henn. & E. Nyman) E. Horak**	**Vietnam**	**LE 262934**	**OQ779469**	MH190183	MH190155	[7], this work
*E. panniculus* (Berk.) Sacc.	Australia	G1680	—	MK278012	—	[54]
*E. percoelestinum* O.V. Morozova, Noordel., Vila & Bulyonk.	Spain	LE 254390, holotype	KF745927	KF745928	—	[39]
***E. peristerinum* O.V. Morozova et T.H.G. Pham**	**Vietnam**	**LE F-343653, holotype**	**OQ779466**	**OQ804522**	**OQ779188**	this work
***E. peristerinum***	**Vietnam**	**LE F-343649**	**OQ779468**	—	—	this work
***E. peristerinum***	**Vietnam**	**LE F-343650**	**OQ779467**	**OQ804524**	**OQ779186**	this work
***E. peristerinum***	**Vietnam**	**LE F-343652**	—	**OQ804525**	**OQ779187**	this work
***E. peristerinum***	**Vietnam**	**LE F-343651**	—	**OQ804523**	**OQ779189**	this work
*E. placidum* (Fr.) Noordel.	Sweden	UPS:BOT:F-121714, epitype,	KC898394	KC898514	—	[39]
*E. plicatum* (Largent) Blanco-Dios	USA	DAR: DLL10091	—	—	MG702627	[7]
*E. prunuloides* (Fr.) Quél.	USA	AFTOL-ID: 523 4765 TJB	—	—	DQ457633	[55]
*E. quadratum* (Berk. & M.A. Curtis) E. Horak [*Inocephalus quadratus* (Berk. & M.A. Curtis) T.J. Baroni]	Russia: Far East	LE254355	KC898452	KC898504	—	[39]
*E. quadratum*	USA: New York	CORT: TJB 8214	—	—	MH190162	[7]
*E. sericellum* (Fr.) P. Kumm. [*Alboleptonia sericella* (Fr.) Largent & R.G. Benedict]	Russia: Caucasus	LE 254362	KC898453	—	—	[39]
*E. sericellum*	USA: California	HSU: DLL9524	—	—	MG702617	[7]
*E. sericellum*	Belgium	MEN 200315	—	GQ289190	—	[5]
*E. serrulatum* (Fr.) Hesler	Russia: Caucasus	LE 254361	KC898447	KC898501	—	[39]
*E. sublaevisporum* Vila, Noordel. & O.V. Morozova	Spain	LIP JVG 1070823T, holotype	KC898436	KC898518	—	[39]
***E. tadungense* O.V. Morozova et T.H.G. Pham**	**Vietnam**	**LE F-343680, holotype**	**OQ779470**	—	—	this work
***E. tadungense***	**Vietnam**	**LE F-343681**	**OQ779471**	**OQ804526**	—	this work
*E. tibiiforme* (Largent & Aime) Blanco-Dios [*Trichopilus tibiiformis* Largent & Aime]	Guyana	MCA2426, holotypus (BRG, LSUM)	—	—	MG702645	[7]
*E. tjallingiorum* Noordel. var. tjallingiorum	Sweden	UPS:BOT:F-016378, holotype	KC898412	KC898509	—	[39]
*E. turbidum* (Fr.) Quél.	Slovakia	MEN 200351	KC710060	GQ289201	—	[5]
*E. umbrophilum* Noordel. & Hauskn. [*Leptonia* *umbrophila* (Noordel. & Hauskn.) Largent]	Australia: Queensland	DLL9766 (BRI, CNS)	—	—	MG702638	[7]
*E. undatum* (Gillet) M.M. Moser	Russia: European part	LE 312417	MF476910	MF487801	—	[42]
*E. venustum* Wölfel & F. Hampe	Germany	L, Wö E17/10, holotype	KC898355	KC898523	—	[39]
*E. vernum* S. Lundell	Russia: European part	LE 312538	OL338282	OL338537	OL405539	[38]
*E. violaceozonatum* Noordel. & Liiv	Estonia	L 275, holotype	KC898448	KC898502	—	[39]
*E. virescens* (Sacc.) E. Horak ex Courtec. [*Inocephalus virescens* (Berk. & M.A. Curtis) Largent & Abell-Davis]		MEL:2379813	MF977981	—	—	Genbank
*E. virescens*	Guyana	MCA 2479	—	GU384622	MG702629	[7]

## Data Availability

The DNA sequence data obtained from this study have been deposited in GenBank NCBI (https://www.ncbi.nlm.nih.gov/genbank/, accessed on 13 April 2023).
